# Readiness and implications for adopting digitized payment of community health workers: a qualitative study of Benin and Uganda

**DOI:** 10.3389/frhs.2025.1633392

**Published:** 2025-11-18

**Authors:** Joan P. Kabayambi, Kéfilath Bello, Angela N. Kisakye, Vanessa D. Sekpon, Christelle Boyi-Hounsou, Juliet Aweko, Elizabeth Ekirapa Kiracho, Peter Waiswa, Géraud Padonou, Sam Agatre Okuonzi

**Affiliations:** 1 Department of Health Policy, Makerere University School of Public Health, Kampala, Uganda; 2 CNHU-HKM Centre de Recherche en Reproduction Humaine et en Demographie, Cotonou, Benin; 3 Department of Research, Ministry of Health Coutinou Benin, CouCoutunou, Benin; 4 Arua Regional Hospital, Ministry of Health Uganda, Kampala, Uganda

**Keywords:** digital health payment, Africa south of the Sahara, immunization, community health workers, gender, readiness, protocols and systems

## Abstract

**Introduction:**

Community health workers (CHWs) provide lifesaving services to underserved and rural populations. However, CHWs face difficulties in receiving payment, which causes dissatisfaction and loss of motivation in their work. Digital health payments (DHPs) offer some solutions but there is a gap in knowledge and experiences in using DHPs in Africa. The study aimed to assess the countries' readiness to adopt DHP, and develop DHP adoption protocol and strategy.

**Methods:**

This was a qualitative study based on a literature review, key informant interviews, in-depth interviews and a thematic analysis. Forty-nine interviews were carried out with participants consisting of CHWs, MNOs, managers/supervisors, and payers. A thematic analysis provided information on the readiness for DHP at individual, institutional and national levels. A protocol and strategy for adoption were imputed from the data.

**Findings:**

Direct literature on DHP was scanty. Instead, literature linked DHP to PHC, health financing, digital technology and digital economy. Respondents acknowledged the convenience of digital payment. CHWs observed the delay in digital payment, and the prolonged registration and processing. CHWs reported variations in payment and many cases of non-payment. All respondents expressed concern about the lack of systems for complaints. CHWs admitted that there was considerable digital illiteracy among them. Women appreciated their independence and privacy of financial dealings using DHP. No significant gender differences were reported in digital payment but significant differences were reported in literature. Readiness for DHP was rated high for DHP in both countries in organization, infrastructure and competences, but low in procedures and communication. Readiness in legislation, policy, digital infrastructure, and leadership, was rated medium in both countries. However, Benin showed a higher national-level readiness in political leadership and communication, while Uganda demonstrated higher levels of individual awareness, knowledge, and acceptance. To adopt and operationalize DHP in the two countries, the protocol identified essential inputs, processes, outputs, and outcomes, and cross-cutting elements of gender, leadership, policy and public awareness. Four broad strategies were imputed to support DHP: 1) promoting digitized PHC, 2) common national system for financing of CHWs, 3) integrated and interoperable systems, and 4) uniform payment of CHWs.

**Conclusion:**

Although Benin and Uganda are at relatively different levels of readiness to adopt DHP, both countries reported similar experiences. Both countries have embraced DHP with positive policies, but major challenges remain in systems, digital knowledge and infrastructure. The two countries are transitioning to digital economy, which requires integration, interoperability and digitization of PHC and DHP. Common and harmonized systems for PHC financing need to be set up. As a major workforce in PHC, a deliberate effort is needed to improve CHWs motivation and performance through commensurate, safe and timely payments. Each country should design a manual to guide the adoption and operationalization of DHPs nationwide.

## Introduction

### Background

Community health workers (CHWs) are of paramount importance in bringing lifesaving services to those who need such services the most (1–4). One crucial service CHWs provide is frontline implementation of mass health campaigns, including immunization (5). However, countries face challenges of retaining CHWs because of their low pay on the one hand (5, 6) and the challenges they face in receiving their payment, on the other. These challenges include exploitation by some supervisors, long journeys or prolonged waiting times to collect payments, and unreliable payments (7–9). Moreover, when CHWs and those paying them are not in the same place location, it necessitates travelling with large amounts of cash which is risky (7). In addition, cash payment to frontline workers during mass health campaigns presents other significant operational challenges (4, 10, 11).

Digital payment systems are widely used globally but are still in early stages in Africa for paying CHWs. Digital payments facilitate an efficient, secure and transparent payment system. Most African countries are now experimenting digital payments for frontline workers. Digital financial services (DFS), enable financial transactions via mobile phones, the internet, or bank cards, offer a promising solution to these challenges. DFS facilitate efficient, secure and transparent transactions (12). They also have the potential to reduce financial transaction costs (7) and improve access to formal financial services, especially for users and beneficiaries in remote areas (13).

DFS in the health sector are referred to as digital health payments (DHPs). These are electronically facilitated payments made to health workers. DHPs are known to save time and reduce costs by minimizing the need for physical payment by cash. They also help curb theft by directly transferring funds from financial institutions to beneficiaries, bypassing intermediaries who could divert funds (7, 14). Moreover, DHPs have been shown to improve the motivation and satisfaction of CHWs, leading to better outcomes in the health campaigns they carry out. Various digital platforms, such as mobile money, bank transfers, debit cards, and electronic money, are used for DHPs (15).

Due to these benefits, DHPs are increasingly being adopted to pay CHWs and other frontline workers in mass health campaigns, particularly in immunization (13). Successful adoption of DHP in Africa has been reported in Burkina Faso, Democratic Republic of Congo, Gambia, Liberia, Nigeria, Republic of Congo, Sierra Leone, Somalia, and South Sudan (10, 16). In collaboration with WHO, donors, and national governments, and in partnership with local banks and mobile telephone companies, CHWs have been paid successfully (10). In fact, DHP is the only payment method available in politically fragile states such as Somalia where conventional financial systems are unavailable (17, 18).

However, DHPs for CHWs are still designed and implemented in a fragmented way, and large-scale use of DHPs in sub-Saharan Africa remains limited (19, 20). Besides, experiences of governments and national agencies in adopting and scaling up DHPs for CHWs are hardly known. Real-life experiences of CHWs and other beneficiaries using DHP system are scarcely described in literature, leaving gaps in knowledge. Additionally, there is little insight into the nature and levels of readiness and capacity of national stakeholders to adopt DHP systems.

Addressing these gaps is critical for enabling African countries to effectively leverage DHPs, ensuring equitable, timely, and transparent payments for CHWs, and in improving the overall impact of primary health care campaigns (21–23). Increasingly, donors and governments are mandating the use of DHPs as a standard method of paying CHWs (24–27). Consequently, it is crucial to gather in-depth information about how DHP systems function, the challenges encountered, best practices and the readiness and strategies to build sustainable DHP payment systems for CHWs and other frontline workers.

### Country contexts

#### Benin

Benin is a francophone West Africa country with an estimated population of 13 million in 2023. The formal health system in Benin pyramidal with three at national, regional and district levels. The national level consists of Ministry of Health and its specialized agencies. The regional level is made up of health directorates. The district level is the most decentralized level of the health system, characterized by its proximity to the grassroots population. This healthcare system is complemented by a huge network of CHWs, acting as a bridge between the population and the health system.

Historically, the hiring, training, activities, and payment of CHWs were fragmented. However, since 2018, several organizations—including Catholic Relief Services, WHO, and UNICEF—began using mobile money for payments during immunization and health campaigns (14). From 2020, mobile payment of field workers during immunization campaigns was put under the Ministry of Health. The national malaria control program (NMCP) launched Benin's first fully digitalized long-lasting insecticidal net distribution campaign, which included mobile payments for field workers (25, 28). This was followed by the digitalization of a malaria chemoprophylaxis campaign in northern Benin. The country has since embarked on a broader effort to digitalize public services, as part of its 2021 national digital economy strategy, which focuses on e-governance, e-education, and e-commerce (26, 27).

#### Uganda

Uganda is located in East Africa and has a population of about 46 million. The country has embarked on a concerted journey to digitize payments of CHWs in the country (24, 29). This has been through public-private partnerships with civil society organizations and private entities. Uganda launched a 5-year National Community Health Strategy in February 2023 followed by the Uganda Health Information and Digital Health Strategic Plan in May 2023.

Uganda has five levels of the national health system: national, regional, district, sub-district and community levels. At national and regional levels are specialized referral hospitals with supportive and outreach services to lower levels. At the district and sub-district levels are general hospitals with large community outreach services. At community level, CHWs play a big role alongside the formally employed health staff.

In 2018, the government, with support from various partners, rolled out a mobile money payment system for CHWs working under the Village Health Teams (VHTs) program. This initiative aimed to ensure timely and secure payments, improving motivation and retention of CHWs (28). A broader program of financial inclusion started with the partnership between a bank and mobile network operator in 2009 (1, 29).

Table 1 highlights key features of the two countries: geographic location; existing policies on Primary Health Care and community health; existing policies regarding DHP; profile of CHWs; mass health campaigns often carried out in the country; institutions that organize these campaigns; when DHP started for CHWs; whether digital payment is an isolated intervention for CHWs or is widely adopted for various financial transactions in the country; whether DHP is systematically used to pay CHWs and other health workers, or it is used only in specific experiments or specific projects; and what the mobile money penetration in the country is (proportion of people using mobile money in the country).

**Table 1:** Comparison of country context: Benin and Uganda.

Key features	Benin	Uganda
Location	Francophone West Africa	Anglophone Uganda
Policy on PHC and community health	National Health Policy Community Health Policy	National Health Policy Community Health Strategy
Policy on DHP	Digital Economy Strategy	Health Information and Digital Strategic Plan
Types of CHWs	Community relays (untrained CHWs) Qualified CHWs (formally trained)	Village Health Teams (untrained) CHWs (untrained) Community Health Extension Workers (CHEWs) (new cadre to be trained)
Training of CHWs	Untrained CHWs are for specific tasks only Qualified CHWs are trained up to 3 years	VHTs and CHWs not formally trained CHEWs to undergo formal training
Remuneration of CHWs	CHWs paid per task about USD 83.5/month Qualified CWHs are paid as per labor law	CHWs payment is not specified CHEWs are paid about USD10/month In addition, they get allowances related to tasks given.
Health campaigns involving CHWs	Immunization Seasonal malaria prophylaxis Insecticide Treated Net distribution Onchocerciasis drug distribution	Child health Immunization Covid-19 immunization Ebola surveillance Cancer awareness campaigns Malaria campaigns
Major partner organizations	WHO Unicef CARE USAID	WHO Unicef CARE USAID UKAID AFENET
When did DHP start?	2018 as a scaled-up program, but smaller experiments started much earlier	In 2018 mainly for CHEWs facilitated by UNEPI through the Ministry of Finance. Other smaller DHPs by partners started earlier.
Organizations that use DHP	MOH agencies and programs Donor partners and NGOs	UNEPI/MOH also supporting other programs and departments All key partner organizations
Is DHP widely used or is it isolated?	DHP is part of digitization of community health services Part of wider digitization of public service	DHP part of the wider strategy for e-governance
Is DHP systemic, experimental or *ad hoc*?	Initially used for specific, stand-alone campaigns, now being made systemic	Initially used for specific, stand-alone campaigns, now being made systemic
Mobile phone penetration	67.3% in 2023 (Source: Electronic Commission Regulatory Authority)	67.7% in 2023 (Source: Finscope Survey)
Mobile money penetration	44.51% 2023 (Source: Electronic Commission Regulatory Authority)	Estimated at 47% in 2023 (Source: World Bank)

### Research justification and objectives

The key research question is: how ready are Benin and Uganda to adopt digital payment of health workers? And what are the implications for adopting DHP? Therefore, the aim of this study was to assess nationwide readiness to adopt digital payment systems for CHWs and other frontline workers. The study objectives were to understand the experiences of CHWs and payers of DHP, to assess readiness to adopt digital payment systems nationwide, and to design a protocol and strategy to adopt digital payment systems for CHWs and other frontline workers.

## Methodology

### Literature review

Digital payment CHWS is a recent development. Not much is known or written on it. Thus, as part of the methodology, we sought to understand DHP in a wider context. Literature review guided us to develop the rest of the methodology. We searched and reviewed both general literature and official documents. We made web searches in PubMed, Scopus, and other social science databases using the keywords:

- “Digital Health Payment” AND “Africa South of the Sahara” AND “Immunization”
- “Digital Health Payment” AND “Africa South of the Sahara” AND “Community Health workers”
- “Digital Health Payment” AND “Africa South of the Sahara” AND “Gender”
- “Digital Health Payment” AND “Readiness”
- “Digital Health Payment” AND “protocols or systems”

We also searched documents on websites of organizations practicing DHP for CHWs or for immunization campaigns. We collected documents from organizations cited above and from the governments of Benin and Uganda. The search was restricted to ten years, from 2013 to 2023. We obtained 59 papers out of which we chose 38 as relevant for the review. A data extraction format was used to harness information from literature review. The data was collated, synthesized and summarized into tabulated descriptive narrative.

### Study concepts and conceptual framework

The study used two key concepts: Readiness and Appreciative Inquiry (AI). “Readiness” is a social science concept that refers to the preparedness of an individual or group to undertake an action or bring about a desired change (30, 31). An individual's readiness is their level of awareness, acceptance, capability, knowledge and level of training to accomplish a task in order to cause change. An organization's readiness is a shared resolve by its members and stakeholders to implement an agreed or desired change (32–34).

Appreciative Inquiry (AI) identifies alternative problem-solving approaches, leads to designing effective strategic plans, and elaborates step-wise protocols of implementation. It focuses on what works, rather than trying to fix what does not. AI offers an approach for evaluating and envisioning future initiatives based on existing best practices (31). It has four components known as the 4Ds: discovery (research), dream (vision and strategy), design, and destiny (sustainability and scaling up). It helps to identify inputs, processes and context of experiments, so as to discover factors for successful adoption, scale-up and sustainability of programs (32, 33).

**Figure 1:**
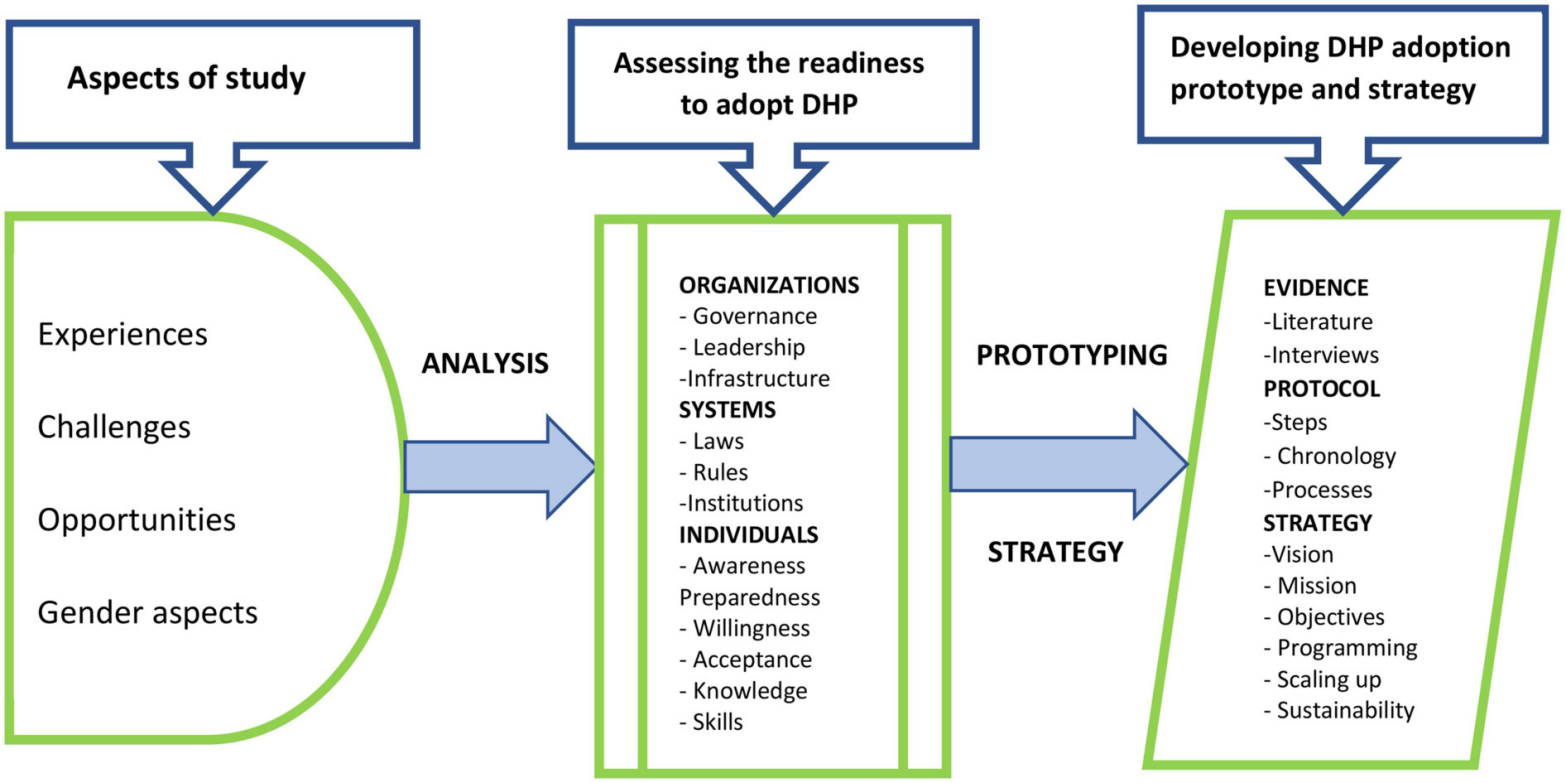
Conceptual framework.

The conceptual framework (Figure 1) consists of three parts. Part one has experiences, challenges, opportunities and gender. The second part of the study consists of readiness of individuals, systems and organizations. And the third part is the construction of the adoption protocol and strategy.

### Study design

This was a qualitative exploratory case study. The case was the implementation of DHP, with a specific focus on mobile money payment, and immunization and other mass health campaigns. Mobile payments are the most widely adopted form of DHPs in African countries.

### Study setting

The study was conducted in Benin and Uganda, chosen for ongoing DHP initiatives and contrasting contexts in terms of geographic location (West and East Africa, respectively), socio-economic and cultural differences and different stages of nationwide DHP. In Benin, the research was carried out in two health districts and the capital city, where key national institutions are based. In Uganda, the study was done in two health districts, urban and peri-urban, close to the capital city. Easy access, cost and convenience were considered in the selection of sites.

### Study participants

While CHWs provided in-depth information about DHP, other categories of respondents provided information to mitigate against CHWs' possible recall bias. These were supervisors, managers, payers, and GSM/MNO operators. In addition, these categories of participants provided other specialized information which only they could provide. Altogether 49 interviews were held. These 49 participants were selected as follows: at least one CHW from each location, at least one GSM/MNO manager from each country, at least one payer from each of the different organizations or programs, and at least one national supervisor or manager. Table 2 below shows the different categories of participants interviewed.

**Table 2:** Categories of participants.

Categories of participants	Benin	Uganda
CHW	12	15
GSM/MNO	2	0
Payer	6	1
Program/Organization	2	6
National Supervisor/Manager	3	2
Total	25	24

### Sample size and sampling

We selected two organizations or programs per country. These organizations were purposively selected because they were already practicing DHP, and were readily available. The main selection criterion for the study participants was their involvement in DHP process or being a beneficiary of it. In Benin we selected the national agency for primary health care, which is responsible for immunization and malaria. In Uganda, organizations selected were Uganda National Expanded Program on Immunization (UNEPI), Family Health International (FHI 360) and Living Goods. Under them were a range of partner institutions which provided study participants.

As much as possible equal numbers of male and female respondents were selected, to obtain a gender perspective. Through a pre-visit, managers, supervisors, digital service providers, and payers were identified prior to the main study. CHWs who had been paid at least twice using DHP system were selected, balancing CHWs from urban areas with those from rural areas. CHWs who were in active service and those who had left the service were also selected for comparison.

### Data collection

Key informant interviews (KIIs) were conducted with national and district managers, Mobile Network Operators (MNOs), payers and supervisors. Respondents were chosen purposively, based on expertise in DHPs for CHWs. In-depth Interviews (IDIs) were conducted with CHWs. In total, 49 interviews were conducted: 27 IDIs (12 in Benin and 15 in Uganda) and 22 KIIs (13 in Benin and 9 in Uganda).

Interview questions were on DHP process, respondents' awareness of DHP, and their preparedness, willingness, acceptance, knowledge, capability and training related to digital payments. We probed for answers on infrastructure, political will and support, regulation, the digital ecosystem, plans for expansion and sustainability, existing payment policies; and benefits, opportunities, benefits and challenges of DHP.

### Data analysis

Recorded interviews were transcribed into Microsoft Word by qualified transcribers. Data was exported and coded. A thematic content analysis approach (35) was used to analyze relevant and important themes. Themes were coded and classified. A gender-lens was also used throughout data analysis to identify gender specificity in users' experiences, challenges and readiness for DHP, and to determine gender-specific actions needed in a DHP system. Readiness was analyzed at three levels: individual, organizational, and national.

Researchers rated readiness as high, medium or low, based on the level of perceived performance, the level of need for improvement, and severity of challenges. High readiness was when a dimension was functioning well, with little to no need for improvement. Medium readiness was when positive aspects were identified, but with room for improvement. Low readiness was when significant challenges were present, requiring substantial improvement. See Table 3. Based on the findings, a framework was made to help plan how to transform current DHP practices into a national, inclusive and sustainable digital payment systems.

**Table 3:** Framework for researchers’ rating of a country's readiness to adopt DHP.

Levels	Rating	Dimensions considered
1	High	Dimension already working well Minimal challenges Little or no need for improvement to adopt DHP
2	Medium	Many positive aspects of the dimension Significant challenges Considerable room for improvement
3	Low	Dimension not functioning or available Significant associated challenges Substantial improvement needed

## Findings

### Literature review

Table 4 presents a synthesis of key findings and references from literature. DHP was mentioned under five themes: primary health care (PHC), CHWs, health financing, digital economy, gender and digital payments, readiness for DHP and DHP systems.

**Table 4:** Literature review findings and references.

Themes	Sub-themes	Key messages	References
CHW Programs	PHC Health Financing Universal Health Coverage	Digitized payment increases transparency and provider satisfaction 78% of CHWs are satisfied with DHP DHP enablers are: technology and willingness CHW performance depends on reliable payment system Successful CHW programs depend on a strong PHC International Labor Organization (ILO) makes it imperative for all CHWs to be paid regularly. People centered health financing has 4 attributes: public funded, pooling, equity and based on people's needs.	Agarwal S, Abuya T et al. 2021 Ekirapa E, et al. 2022 Mugisha D 2022 Mangone et al. 2021 HFG 2015 Cometto G, Ford N, et al. 2018 Athanase C, Hounnakan A, et al. 2021 Ballard M, et al. 2021 Governments and Partners 2015 Hanson, Brikci N, et al. 2022 McConnel et al. 2022 WHO 2018 Hamani A et al. 2023 Benin (Government) 2023 GOB 2022 Zheng C, et al. 2019 Yehualashet et al. 2016 WHO 2018a Wemos and Achest 2019 Pandya S, Hamal M et al. 2022 Ormel, Kane S, et al. 2019 O’Brien N Li E, et al. 2022 Lankester T 2019 Kallon I 2020 Hemily D et al. 2021
Digital payments	Cashless economy Financial inclusion Digital Infrastructure Digital technologies	Global push for cashless economies. Key pillars: e-governance, e-education and e-commerce In digital payments women are hindered by limited access to phones, to salaried work, and by low economic status Challenges of digital payments: unreliable internet and power, delays in payment, high charges, variations in payee details, lack of feedback, fragmented legal framework Opportunities: high political will, a variety of banking services, increasing digital economy	Government of Benin 2020 AFI (Alliance for Financial Inclusion) 2019 HFG 2015 Egami H, Matsumoto T 2020 Huang et al. 2017 IFC 2018 Gentilini U et al. 2021 Tay, Tai and Tan 2022 Vital Wave 2014 Mugisha D 2022 Muhangi, Mullengi et al. 2022 Yehualashet et al. 2016 WHO 2018b Sessions, Gatt et al. 2014 Soutter, Ferguson et al. 2019 Rohatgi, Galdava et al. 2019 Gronbach L 2020 Lugada E, Komakech H et al. 2022 Labrique et al. 2018
Readiness for DHP	Institutions/systems Infrastructure Adoption protocol	Peer learning is imperative Synergy, synchrony and interoperability of technologies Dispute resolution systems Network coverage and reliability Steps for adoption of DHP are: planning, contracts, payment system, account procedures, registration of payees, testing system on staff, testing on beneficiaries, and scaling up	Weiner, B J 2009 Shuayb M, Sharp C et al. 2009 Shea, Jacobs et al. 2014 Benin Ministère de la Santé Publique, Bénin 2015 Holt, Armenakis et al. 2007

Here are the key points in literature. Digital payments are reported as increasing transparency and provider satisfaction. This is reflected by 78% of CHWs who are satisfied with DHP. DHP is enabled mainly by technology and by the willingness of people: leaders, payers and payees. CHW performance in their work depends on the reliable payment systems. Successful CHW programs also depend on a strong PHC system. Currently a good number of CHWs are volunteers in PHC, but they cannot always be relied on. Yet, International Labor Organization (ILO) has made it imperative for all CHWs to be paid regularly, in compliance with international labor law. Payment and non-payment of CHWs is dependent on available financing, governed by public policy. The new approach to revitalized PHC proposes a people centered health financing. This type of financing has 4 attributes: public funding, pooling of funds, equity and based on people's needs.

Currently, there is a momentum of a global push for cashless economies. The key pillars of a digital or cashless economy include e-governance, e-education and e-commerce. In digital payments, women are often hindered by limited access to phones, to salaried work, and by low economic status. The common challenges in digital payments include unreliable internet and electricity, delay in payment, high charges, variations in payee details, lack of feedback, and fragmented legal framework. However, there are opportunities for establishing and improving DHPs. These include high political will, a variety of banking services, and increasing digital economy.

Peer learning is imperative in a rapidly changing technological world. Synergy, synchrony and interoperability of technologies are critical. There is need for dispute resolution systems in digital payments. Network coverage and reliability are critical. To adopt DHP the following generic steps have been proposed: planning, making contracts with key players, elaborating the payment system, establishing accounts management procedures, registration of payees, testing the system on the staff, testing the system on beneficiaries, and then scaling up.

### Thematic analysis of interviews

Experiences generated from CHWs were collaborated by network operators, program managers/supervisors, and payers. Themes generated from qualitative data analysis were as follows:

1. Convenience of digital payment
2. Delay in digital payment
3. Variations in payment and non-payment
4. Lack of a system for complaints
5. Digital illiteracy
6. Prolonged registration and processing
7. Women's appreciation of their independence and privacy by using DHP

#### Convenience

Most CHWs, in both countries, knew and appreciated the trend of payment of their allowances or wages by mobile money. They were aware that cash payments were being phasing out everywhere. They knew the requirements for digital payment included identification with corresponding names on NID and payment register, and the need to authorize the use of another person's names and sim card, when necessary. CHWs knew the practical process of digital payment. Below are some of the responses:

“When you receive money on your mobile phone, as long as you don't tell the person you don't have to give money to anyone. […] Digital payment is a very good thing. It saves us travel costs and the hassle of sometimes being asked to come to [a place] and pay [in cash]. In that respect, it's a good thing”. Female CHW, Benin

“Mobile money is convenient, we work in the same district and payment comes to us in same place, no transport cost” Female CHW, Uganda

“…mobile money is reliable. It saves time, saves on transport cost. I save my money on the phone, when it has accumulated, I send it to the bank” Male CHW, Uganda

“Today, when you look at everything that's going on in the country, it's the best. It's the best means of payment. Firstly, it's a fast payment, you make sixteen thousand payments in ten minutes, sixteen thousand paid in ten minutes, you no longer carry the funds to the most remote areas, the transaction is done from the bank to the MTN account opened and so the electronic money is positioned, and the transfer is instantaneous.” Male GSM key informant, Benin

#### Delay in digital payment

Several reasons were cited by the payers and CHWs, to explain delays in payment. These included: non-conformity of CHWs' personal information across various sources, usually because of a wrong entry by mistake of names and telephones numbers of CHWs at registration. Lengthy organizational procedures: these include the time needed for checking and verifying payment statements. In addition, there were several levels of security checks, which payers insisted, were necessary. Other reasons were the long processing times by payers when errors occurred, and the slow processing and transmission of documents by MNO operators during the validation. There were inefficient communication and feedback among the various actors which slowed the payment process.

Here is what CHWs said corroborated by a MNO key informant:

“Let's say we have worked in November and you get paid in January or even not get paid at all. For example, we, who give out drugs in ICSEM, it takes long for us to get paid.” Female CHW, Uganda

“Nowadays what still hurts…is the delay in payment. Otherwise, you've got your own account and that's fine. The delay in payment… That's what hurts”. Male CHW, Benin

“By the time someone has done an activity, they should have been sensitized that their phone number and name should correspond. Some of them wrongly fill in the form and they don't get their money and they keep calling the coordinator. Yet the problem is with the person” Male MNO Key Informant, Uganda

### Variation in payment and non-payment

In most cases, beneficiaries received full payment. In some cases, CHWs did not receive the full amount they were expecting. There were also perplexing and unexplained variations in payments, after having done the same amount of work. In other cases, beneficiaries did not often receive their payment at all. Some CHWs would get unexplained payments. Sometimes CHWs don't tell the truth about receiving money. Here are some of the responses:

“The challenges are that at times when we are promised to be paid, we don't get paid. You wait for money after the activity and you never get it. You just forget about it”. Male CHW, Uganda

“Just recently some people received Shs32,250 but nobody, including myself the coordinator, could remember for what purpose was this money paid” .Male CHW, Uganda

“I hear people say that they have received less than they should have […]. I don't know if it's true […] because there are some people who receive and say they haven't received anything. You check and you see that the money is inside, so you can't say that what they're saying is always true”. Female Supervisor/Program Manager Key Informant, Benin

#### Lack of systems for complaints

There was lack of a system to respond to complaints, compounded by lack of communication between payers and payees. Indeed, a common challenge everywhere was that once you missed payment, it was almost impossible to reclaim it. CHWs were mindful of the voluntary nature of their work and that they had very low pay. With DHP, even this low pay can become unpredictable and sometimes not paid at all.

Here is what a CHW said:

“….during immunization program, you go to work, and you do not know when your money is coming. You borrow money hoping to be paid and end up in bad books with neighbors. This is not acceptable. We have lost relations”. Male CHW, Benin

#### Digital illiteracy

In Uganda, there were cases of being defrauded by mobile money agents. There were also cases of digital illiteracy whereby CHWs forgot their passwords or pin numbers and needed assistance. This assistance was paid for out of the meagre pay of the affected CHW.

“Almost 55% of community members don't know how to use mobile money. So, I recommend that if there is a chance to train people in the usage of mobile money” Male CHW, Uganda

“They will easily get conned. The mobile money agent can ask them for their PIN number and if they give it in, the agent can withdraw money from your account without your knowledge”. Female CHW, Uganda

#### Prolonged payment process

Lengthy organizational procedures, which include checking and verifying payment statements. In addition, there were several levels of security checks. There are many requirements for identification with corresponding names on the National Identity Card (ID) and payment register. There is often the need to authorize the use of another person's names and sim card, when a payee does not have a sim card. When an error occurs and the process is severely delayed. There is also the slow processing and transmission of documents by MNO operators during the validation phase.

Here is what a CHW said:

“…When it is from Momo (mobile money), it takes time. You have to double-check the names, and numbers. If it is the right name written correctly on the card, and you have checked properly, you get your money. I know some people whose names are different on the card and in the register, they don't get paid” Female CHW, Benin

This was confirmed by program manager:

“It's true that there can be failures, and when there are failures, it can take time to make claims. When you're entering, for instance, 95 and you mistakenly enter 85, that will be an error. But in *any case, before making the payment, MTN checks it. We send the electronic file to MTN so that MTN can confirm that the numbers are really the same*”. Male Supervisor Key Informant, Benin.

“You can sign the same report more than four times. For a round trip, you pay 1,000 francs for the journey to go and sign. Three or four days later, they call you again to tell you to come and sign another statement because someone made a mistake on the old one and if you don't come and sign, don't blame them afterwards if you don't get your money…” Female Manager Key Informant, Benin

#### Gender differences in DHP

Most respondents said that there was not much difference in digital payments between women and men. However, on further enquiry, respondents revealed some gender-related benefits to women. Mobile payments enabled women to get personal identification documents and mobile money numbers, which could facilitate them to access loans. Mobile money increased women's self-efficacy, self-esteem, and self-affirmation.

A respondent had this to say:

“Yes, it [DHP] makes us financially independent. Because we say to ourselves, this is our own money, it came into my account, I will do what I want with it… When I have money on my mobile, I am confident, and flexible on how I spend the money” Female CHW, Benin

### Infrastructure, systems and policy

The inadequate or absent infrastructure, systems and policy was captured from a Benin government official as follows:

“Regarding the implementation of the national e-health strategy in Benin, the country faces a low rate of ICT usage hindering access to e-health services due to high cost of connectivity. There is instability and low coverage of electrical energy especially in rural areas which makes it difficult to progress in tele-medicine, computerization of hospital structures and mobile health. The incomplete codification of medical acts particularly in e-health is also a challenge. There is a significant deficit of human resources in the public sector which hinders the implementation. The connectivity of health facilities in ICT infrastructure is weak and precarious and there is a delay in implementing the ICT master plan. Additionally, there is insufficient internal skills for ICT infrastructure maintenance” Government official Benin.

#### Other challenges

Other challenges included lack of receipts or acknowledgement of payment. Sometimes CHWs, having done several assignments, had no idea who exactly was paying them and for which assignment. There were complaints about withdrawal charges which ate into the small payment of CHWs. This was addressed by adding an extra amount to cover withdrawal charges. Other challenges were unreliable or absent power supply, and unreliable network and internet. Mobile money agents often lack of cash to pay CHWs.

#### Suggested solutions

Respondents suggested the following solutions to the challenges: 1) training of CHWs on the payment system, 2) reducing payment time to a week, 3) communication and feedback to payees, 4) reducing or eliminating deductions, 5) focusing more on mobile money rather than on bank ATMs, 6) CHWs should all have proper IDs before getting into the digital payment system, 7) organizations should get into proper contracts with CHWs with clear job description and terms of payment, 8) CHW reports should be digital and easily retrievable as proof of work done and to be verified, 9) decentralizing digital payments to local authorities, and 10)sensitizing payers and payees on cyber fraudsters.

Some CHWs suggested a commensurate increase in pay. Here is how one CHW put it:

“If they can increase our remuneration for the work we do, that would be good… You are well aware of the high cost of living that has prevailed for some time. If, for example, we carry out an activity over a period of 10 days, they give us say 15,000 XOF, the remuneration is not commensurate with the effort made” Male CHW, Benin

On opportunities for adopting DHP, here is what a Benin partner organization official said:

“Of course. We're moving towards that [DHP]. All payments, it has already been decided […]. There will be no more sight payments. It's even forbidden […] The opportunities are that many partners are already interested in this, as many of our activities are financed by partners. So, we have funding opportunities. We have opportunities for integration with other sectors that have already tried this. Apart from vaccination, there are impregnated mosquito nets, and there are other programs that do this. So, we have opportunities to use the experience of other programs to do this.” Male, Partner Organization Key Informant, Benin

Table 5 summarizes thematic and triangulation analysis.

**Table 5:** Thematic and triangulation analysis.

Themes	Illustrative CHW Quotes	Related finding from other participants’ quotes or from documents
Convenience of DHP	*“It saves us travel costs and the hassle of sometimes being asked to come to [a place] and pay [in cash]” CHW, Benin*	*“…it's a fast payment, you make sixteen thousand payments in ten minutes, sixteen thousand paid in ten minutes, you no longer carry the funds to the most remote areas, the transaction is done from the bank to the MTN account” GSM Manager, Benin*
Delay in payment	*“Let's say we have worked in November and you get paid in January or even not get paid at all” CHW Uganda*	*“Some of them wrongly fill in the form and they don't get their money and they keep calling the coordinator. Yet the problem is with the person” MNO manager Uganda*
Variation/non-in payment	*“…we are promised to be paid, we don't get paid. You wait for money after the activity and you never get it”.*	*“I hear people say that they have received less than they should have […]. I don't know if it's true […] because there are some people who receive and say they haven't received anything” Program manager, Benin*
No system for complaints	*“The challenges are that at times when we are promised to be paid, we don't get paid. You wait for money after the activity and you never get it. You just forget about it”. CHW, Uganda*	“*There are virtually no rules or regulations, and no redress*” Accountant/payer, Uganda
Digital illiteracy	*“Almost 55% of community members don't know how to use mobile money.”* CHW, Uganda	*“Some of them wrongly fill in the form and they don't get their money and they keep calling the coordinator. Yet the problem is with the person”* MNO Key Informant, Uganda
Variation in/unexplained payment	*“Just recently some people received Shs32,250 but nobody, including myself the coordinator, could remember for what purpose was this money paid.”* Male CHW, Uganda	*“I hear people say that they have received less than they should have […]. I don't know if it's true”. Supervisor/Program Manager, Benin*
Prolonged process	“…*When it is from Momo (mobile money), it takes time. You have to double-check the names, and numbers” CHW, Benin*	*“It's true; there can be failures, and when there are failures, it can take time to make claims”. Supervisor, Benin.*
Gender aspects	“Yes, it makes us financially independent” CHW, Benin “*I once had an account that my husband was aware of. Money would be deposited and then he withdraws it… he tells me that he will pay me back, which is impossible” CHW, Uganda*	“*Significant differences have been observed between men and women in the use of digital financial systems (DFS). The needs, drivers, uptake and patterns of usage of DFS are different between men and women*” (IFC, 2018).

### Views from other participants

While CHWs provided in-depth information about DHP, other categories of respondents provided information to mitigate against CHWs possible recall bias. These were supervisors, managers, payers, GSM/MNO operators.

#### Supervisors

In the mobile payment process, supervisors, who are often head nurses, recruited participants for the various activities requiring mobile payment. Once the CHWs have been trained, supervisors ensured that the attendance lists and payment statements would be re completed by the mobile payment beneficiaries, often CHWs. They would also collect copies of beneficiaries' identity documents and send them on to the Administrative and Financial Director for the rest of the mobile payment process. These are the first link in the mobile payment chain.

#### Payers (indirect and direct)

In Benin, indirect payers are those people involved in preparing mobile payment of CHWs but are not the ones who click the button to make the e-payment. Indirect payers are usually accountants commonly known as Financial Affairs Officers at commune level or Administration and Resources Officer (CAR) at health district level. To prepare mobile payment, indirect payers collect various documents from supervisors to produce digitized payment statements in Excel format. They then pass on these statements to the direct payers for the rest of the payment process.

Direct payers are those people who click on the command enabling the payment to be made, i.e., those who perform the last action for sending the e-payment to beneficiaries. The profile of direct payer varies according to the level of the health system and type of contract established between the organization and mobile service operators. At health district level, direct payers may be a staff of the GSM operator's local agency. At the regional level, direct payers are heads of the planning, administration, and finance department of the regional health office. At the national level, direct payers are accountants of the national agencies implementing health programs that require mobile payment of CHWs.

Direct payers check documents transmitted by indirect payers before payment is made to CHWs. This involves checking the conformity of personal details of the CHWs with the attendance lists, payment statements, and mobile money accounts. They also produce a Comma-separated-values (CSV) file, based on Excel file and the information is provided by the indirect payers.

#### Coordinators or program managers

Coordinators or program managers are usually district medical officers, regional medical officers, or health program managers. The role of coordinators or managers in mobile payment is to issue, before the health activities start, official notes authorizing the mobile payment. Once the preparation for the payment is completed and all documents have been validated by the direct payers, the coordinators or programmers give their agreement in principle before the e-payment is sent to CHWs and other front-line worker.

#### GSM/MNO operators

Global System for Mobile Communications (GSM in Benin) or Mobile Network Organizations (MNO in Uganda) operators are commercial companies investing in the Financial Technology (FinTech) field, among others. The GSM/MNO operators provide mobile payment platforms to organizations implementing mobile payment. The GSM operators also train direct payers to use these platforms. As stated above, GSM/MNO operators can also function as direct payers. In Benin, the GSM operators are Mobile Telephone Network (MTN), Moov Africa, and Celtiis Benin. In Uganda the MNO operators are MTN, Airtel and Uganda Telecom.

In both countries MTN and airtel were the operators preferred by organizations making mobile payments to field workers in vaccination campaigns and other mass community activities. MTN MoMoPay and Ecobank Mass are the digital platforms used in Benin. A digital platform is a user interface that the GSM/MNO operator installs for organizations wishing to make mobile payments themselves, from their offices. Installation of the platform is free of charge, and users receive training from the GSM operator. MTN's platform is the most widely used in the mobile payment experiences implemented by the Ministry of Health in Benin.

### Readiness for DHP adoption

**Figure 2:**
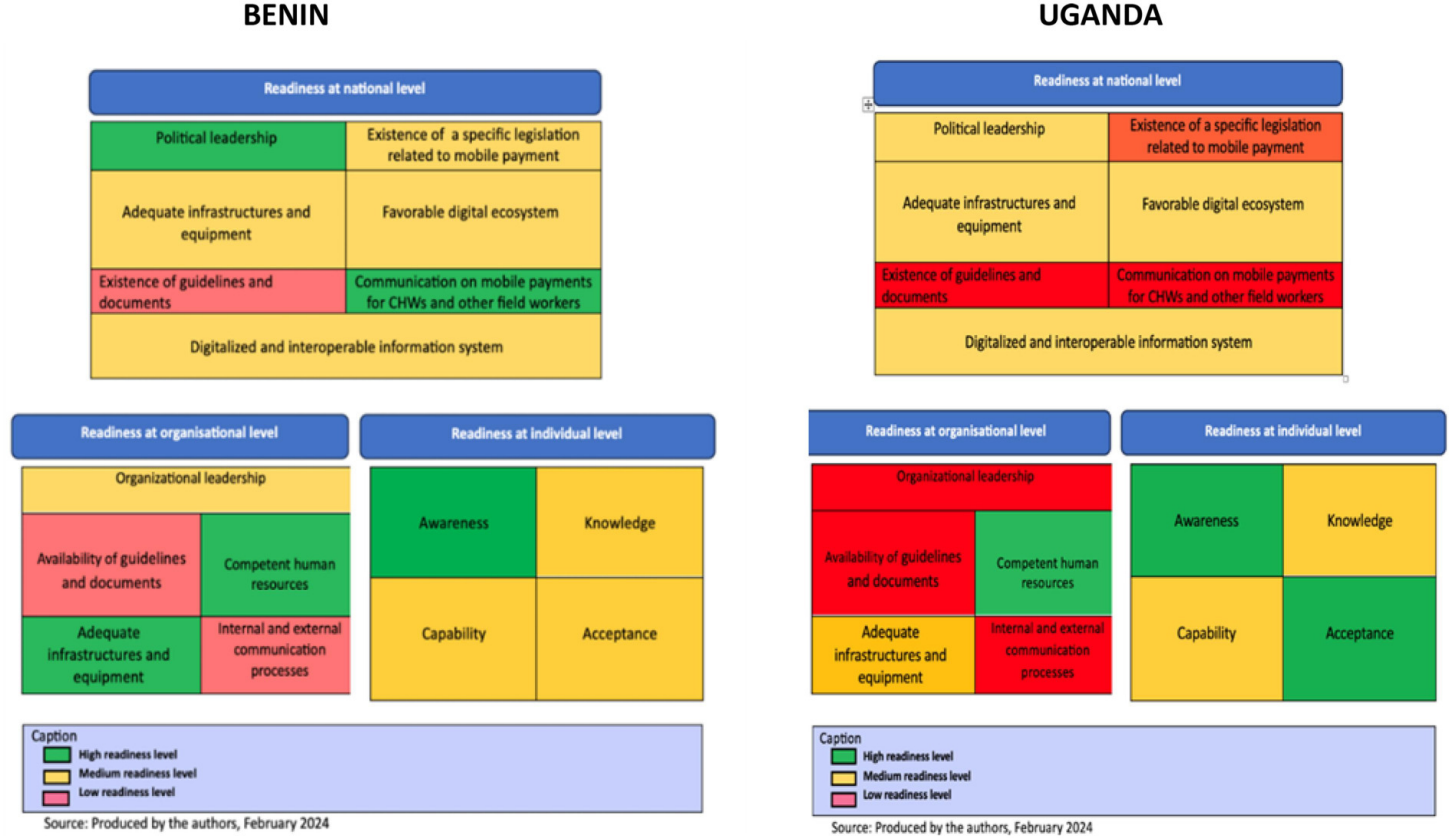
Levels of readiness for digital payment system.

Table 6 and Figure 2 summarize and compare DHP readiness of the two countries.

**Table 6:** Levels of DHP adoption readiness.

Readiness components	Perfect readiness	Actual readiness level in Benin	Actual readiness level in Uganda
Individual level			
Awareness	All CHWs know DHP system exists	High	High
Preparedness	All CHWs and other actors have been sensitized, trained and facilitated	High	Medium
Willingness	All CHWs and other actors express eagerness for DHP	High	Medium
Acceptance	All CHWs and other actors eagerly enroll for DHP without any reservation	Medium	High
Knowledge	All CHWs know the basics of how a digital payment system works All actors know the process well	High	High
Skills	All CHWs can receive and withdraw money from a digital payment system without assistance. All actors are competent to fulfil their roles	High	High
Organizational Level			
Ministry of Health and related organizations	Digital equipment, technical support, policy and regulation exist and are fully operational	Medium	Low
National Level			
Digital infrastructure Digital economic policy and laws	Infrastructure for digital ecosystem covering whole country Technical support is readily available Policy and laws exist and are fully operational	Medium	Medium

#### National readiness

Benin showed remarkable leadership in digital economy. The communication on mobile payments was also assessed to be good. The level of readiness in legislation related to mobile payment was found to be medium. The level of readiness was also judged to be medium for infrastructure and digital ecosystem. In Uganda, four dimensions were rated as medium at the national level: political leadership, infrastructures to support DHP, the digital ecosystem, and the existence of a digitalized and interoperable information systems. Indeed, it was generally acknowledged that Uganda was moving rapidly towards cashless economy, with a strong political stewardship.

#### Organizational readiness

In Benin digital infrastructure and equipment were scored as high. Additionally, organizations had competent human resources. Organizational leadership readiness was rated as medium. There was lack of essential governance tools such as clearly defined procedures and a complaint management system. There was no effective communication system for coordination of actors. In Uganda, organizational readiness was also high for human resource competencies. But was it rated as medium for digital infrastructure. There were major gaps in equipment, technical support, policies and regulations. Readiness in leadership in Uganda was assessed as low due to lack procedural guidelines, and effective communication among stakeholders.

#### Individual readiness

At individual level, awareness and knowledge of mobile payments was good in both countries. All actors were informed about mobile payments and understood the basic requirements for participating in the process.

### Literature synthesis and interpretation

Table 7 presents a synthesis of key findings from literature and its interpretation in relation to DHPs. DHP was mentioned under five themes: primary health care (PHC), CHWs, health financing, digital economy, gender and digital payments, readiness for DHP and DHP systems/protocols.

**Table 7:** Literature review synthesis and interpretation.

Themes	Key findings	Interpretation in relation to DHP
PHC	A strong PHC is the foundation of a resilient health system and economy. But currently PHC is fragmented in Africa, underfunded, weak and not a priority. PHC has many uncoordinated actors.	PHC has to strengthened, integrated, prioritized for funding and digitized
CHWs	60–85% of CHWs in SS Africa are unpaid volunteers, a major disincentive. But CHW services are essential in PHC	CHW payment has to become a priority in PHC
Financing	The most effective PHC financing is people centered consisting of: 1) public funding; 2) pooling of funds; 3) equitable; and 4) responds to community needs	Need for mapping of the national financing ecosystem and formulating a common financing mechanism for all CHWs
Digital economy and digitization	Rapid global trend in financial inclusion, cashless economy and digitization	In Africa, begin careful design of digital payments, integration of financial infrastructure and technologies
Gender	Gender differences exist in DHP in general. However, DHP has enhanced women's financial freedom.	Design digital payment systems that are gender responsive and equitable
Readiness	Individuals have high readiness to adopt DHP but infrastructure, policies and systems are weak or absent	Need for countries to work on infrastructure, policies and systems to adopt DHP as a national payment system
DHP adoption protocol	Best practice requires systematic and phased approach to putting in place DHP inputs and processes, and ensuring desirable outputs and outcomes	A model DHP adoption model protocol needs to be developed for each country at its level of readiness

### Designing a DHP adoption protocol

In designing a protocol for DHP adoption and implementation, we considered inputs from DHP readiness assessment, which are: infrastructure, institutions, training and systems. We also considered DHP establishment and operational processes of the payment system. Finally, we considered DHP outputs of immediate results from process activities, and outcomes as the desired results. The outcomes considered included security and safety of payment, CHW satisfaction and retention, health care coverage and improvement. Cross-cutting elements of the prototype include gender, leadership, policy and public awareness. See Table 8.

**Table 8:** DHP adoption protocol.

Inputs	Process	Outputs	Outcomes
Organizational and readiness Country readiness Operational readiness Institutions, training and systems, Individual readiness Operational readiness (availability of merchants throughout the country to withdraw the amounts requested by CHWs, improved MNO coverage) Awareness of the entire range of procedures and payment deadlines by those involved Raising awareness among CHWs to fully embrace digital payment Willingness of stakeholders to pay a reasonable amount to CHWs CHW supportive policy (e.g., acceptance of digitalized payment statement) Policy on digitized PHC Policy on sustainable PHC and CHW financing National identification cards and registration Databases of CHWCHWs and others beneficiaries other staff including the correct number with the name Training accountant for CHW digital payment mobile money accounts Training of new people for paying CHW to pay CHWs Accountability system for resolving non-payment cases, evaluating Evaluating payment experience Communication between payers, and payees and MNOs M&E mechanism Complaints management mechanism	Establishment of clear and transparent mobile procedures allowing a fast payment Withdrawal charges integration Integration of payment budget Electronic validation of working days Creation of digital payment statements Creation of working days Evaluation of payment, including stakeholder satisfaction Drawing up procedures and governing document Setting transmission and processing timelines Managing claims and complaints Evaluation of work done and health system indicators Creating WhatsApp groups for stakeholders and CHWs for follow-up Decentralization of mobile payments to local authorities	Quick payment (within 7 days) Withdrawal charges are paid to beneficiaries by payers Return evaluation reports Return money if payment fails Safe use of mobile money by CHWs Evaluation used to improve the system	Security of payment Better access to payment Improved access for health workers to financial services (loans etc.) CHW satisfaction CHW retention at work Improved service coverage and quality Improved health sector performance
Cross-cutting aspects: Gender policies Public sensitization and education Digital systems Leadership and political will Data systems Identification data bases			

### Designing a DHP strategy

Based on Appreciative Inquiry's 4Ds (Discovery, Dream, Design and Destiny) an equivalent framework (consisting of Vision, Mission, Goals, and Strategies in the planning terminology) was imputed. A DHP adoption strategy is in Table 9. There is evidence that DHP is desirable and a trending phenomenon. The mission is to improve CHW financing and motivation to revitalize PHC towards universal health coverage. The goal is to set up a system that is digitized, integrated, gender-sensitive and sustainable. The strategies are to digitalize PHC, set up common financing units for CHWs, ensure interoperable technologies across board, and improve CHW pay level and payment system.

**Table 9:** Strategy for adopting DHP.

Appreciative inquiry components	Equivalent terminology in strategic planning	Imputed strategic plan framework
Discovery	Evidence	DHP is desirable and trending Accessible in most areas, is safe and provides confidentiality. Drawbacks are: delays in payment and difficulty in reclaiming missed payments Limited use due inadequate infrastructure, lack of policy and systems Some countries now have adequate readiness to adopt it
Dream	Vision	CHWs are motivated to provide equitable, efficient and acceptable PHC services throughout the country
Design	Mission and goal	Mission: To improve financing, service coverage, integration and digitization of PHC and CHW programs Goal: To motivate CHWs through commensurate, regular and safe payments, through gender-sensitive, integrated and digitized systems
Destiny	Strategy	Strategy 1: Promote renewed, digitally-driven, integrated and pluralistic PHC Strategy 2: Develop a common Financing Support Unit/system for CHWs from government, donor partners, insurance, user-fees, community financing and voluntary services Strategy 3: Develop management systems using technologies that are digital, integrated and interoperable across board, and are responsive to PHC objectives Strategy 4: Develop policy and systems to pay CHWs commensurately, regularly and at similar or uniform rates

## Discussion

The study documents users' perspectives as well as their expected and real experiences. Data from Benin and Uganda enabled us to understand that mobile payment for CHWs and other front-line workers is essentially implemented by the Ministry of Health through other players in the expenditure chain. The main impetus for DHPs came from immunization programs because of their intensity and national spread. Initially, DHP started as an experiment and it is now a learning process. Since its adoption in immunization campaigns, good practices have been developed by the players to tackle challenges. However, no concrete protocols have as yet been developed. The results of DHP have largely been positive: beneficiaries' and managers' satisfaction, reduced risk of handling cash, ease of payment and saving on staff costs, among others.

The study confirms that delayed payment is a significant source of dissatisfaction and loss of motivation among CHWs. This is corroborated by literature (4, 23, 30) which identify no-pay, little pay and overly delayed pay as sources of CHW dissatisfaction and demotivation. In addition, this is compounded by lack of feedback from payers, no system of appeal for lost or non-payment, and lack of uniformity and rationale for payments. Electricity, mobile network and internet did not cover many rural areas and were not reliable. These pose a major setback for DHP. These findings are consistent with other studies (29, 36, 37). This study found lack of a clear and uniform system or protocol for DHP in each country. However, the two countries were found to be relatively ready, given the willingness to adopt DHPs and a favorable policy environment. However, more needs to be done to ensure that the mobile payment system is sustainable and inclusive.

This study has led to the understanding that the payment of CHWs is linked to the wider digital economy, to PHC as a whole, to national health financing, and to digitization of management systems. It has also brought insights on gender and women's empowerment in digital payments. Knowledge and awareness about DHP were found to be high in both countries. We found that DHP has gained wide acceptance in the two countries. This contrasts with the general picture of DHP as portrayed in literature as being isolated and experimental small projects (37, 38).

Readiness for nationwide DHP adoption was found be medium to high. This positive finding was not in literature. Generally, sources such as (37) have treated DHP as small and inconsequential. Yet, this study has found an overwhelming interest and momentum to make DHP national systems, consistent with the mass digital adoption innovations already taking place in Africa (38). Benin and Uganda are gearing to go into full scale national DHP adoption. However major gaps remain to enable DHP systems work. These gaps are in infrastructure, policy and systems. DHP also requires wider reforms in PHC and health financing. In particular, there will be need to digitize and integrate systems, and to link it all to the national and global digital economy.

**Figure 3:**
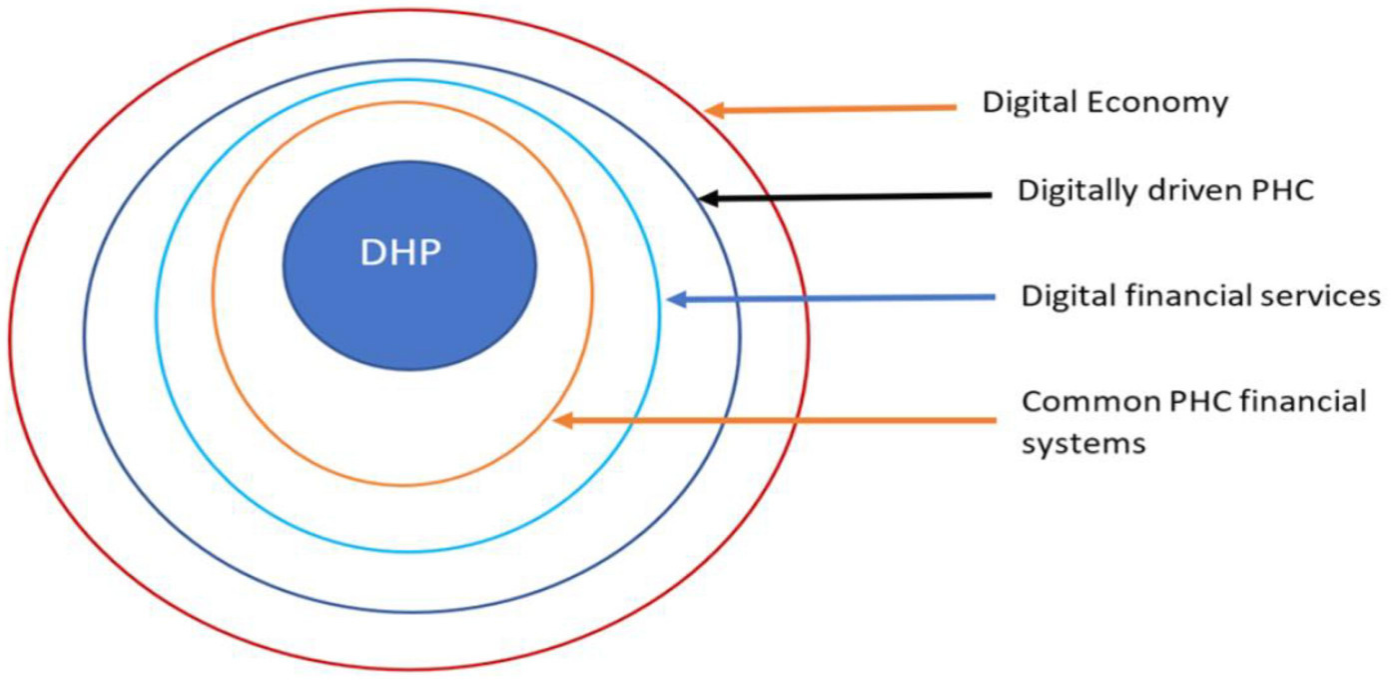
Layers of systems supporting a DHP system.

Therefore, linking all the key elements into a protocol of inputs, processes and systems, we have imputed four strategies: 1) promoting a digitally-driven PHC, 2) developing a common national financing support system for CHWs 3) developing digitally-driven, integrated and interoperable systems nationwide, and 4) a policy to pay CHWs commensurately, regularly, safely, quickly, transparently and uniformly. Figure 3 presents layers of systems that would make DHP system viable.

### Strengths and limitations of the study

One strength of this study is that it enabled us to understand the DHP experiences of CHWs. The value of the study the estimation of the current levels of readiness and in developing frameworks for a protocol and strategy for DHP adoption. The study has provided leads to what needs to be further researched and improved. The limitation of this study is that it was a qualitative study of only two countries. Thus, future studies on CHWs' mobile payment experiences could include direct and measurable observations of the payment process. There is need to corroborate study findings with actual payment records to identify payment failures and successes.

## Conclusion

This study found DHPs already fairly widely practiced in the two countries, in contrast to what literature says. The study also demonstrates a strong will of the two governments and their health partners to adopt DHP as a key move towards digitization of health governance and financial inclusion. Although Benin and Uganda are at relatively different levels of readiness to adopt DHP, both countries reported similar experiences.

The bigger picture from this study is that the two countries are transitioning towards digital economy. This requires interoperability of systems and digitization of PHC. Common and harmonized systems for PHC financing need to be set up. Deliberate efforts need to be made to improve CHW motivation and performance. CHWs require to be targeted as a key health workforce in primary health care.

We therefore recommend that: continuous public awareness and education be conducted on digital payments; CHWs who form a large and essential part of PHC workforce be made a national priority in financing and payment; PHC be digitized and integrated with other systems and technologies; each country formulates a uniform national CHW financing and payment system; and any new payment systems be integrated and be made interoperable with existing systems and technologies.

Furthermore, we recommend: a specific effort be made to ensure gender responsiveness of payment systems; the completion of the establishment of infrastructure of electricity, mobile networks and internet nationwide; and each country design a handbook to guide the adoption and operationalization of DHPs nationwide. The protocol and strategy proposed in this study can come in handy.

## Data Availability

The original contributions presented in the study are included in the article/Supplementary Material, further inquiries can be directed to the corresponding author.

## References

[1] Agarwal S Abuya T Kintu R Mwanga D Obadha M Pandya S Understanding community health worker incentive preferences in Uganda using a discrete choice experiment. J Glob Health. (2021) 11:07005. 10.7189/jogh.11.07005 33763219 PMC7956012

[2] Benin (Government). Evaluation of Community Health Worker Remuneration Schemes Final Report June 2023 [Internet]. (2023). Available online at: https://assets.speakcdn.com/assets/2594/french_chw_compensation_assessment_final_june_2023_reviewed-2023070602544870.pdf (Accessed September 6, 2023)

[3] Governments and Partners. Strengthening Primary Health Care through Community Health Workers: Investment Case and Financing Recommendations: Report. (2015).

[4] Pandya S Hamal M Abuya T Kintu R Mwanga D Warren CE 2022 Understanding factors that support community health worker motivation, job satisfaction, and performance in three Ugandan districts: opportunities for strengthening Uganda’s community health worker program. Int J Health Policy Manag. (2022) 11(12):2886–94. 10.34172/ijhpm.2022.6219 35461208 PMC10105203

[5] Lankester T. Chapter 8: the community health worker (CHW). In: Lankester T Grills NJ, editors. Setting up Community Health and Development Programs in low and Middle Income Settings. Oxford: Oxford University Press (2019). p. 124, 125. 10.1093/med/9780198806653.003.0008

[6] Ballard M Westgate C Alban R Choudhury N Adamjee R Schwarz R 2021 Compensation models for community health workers: comparison of legal frameworks across five countries. J Glob Health. (2021) 11:04010. 10.7189/jogh.11.04010 33692894 PMC7916445

[7] Health Finance and Governance Project. Mobile Money for health compendium. (2015). Available online at: https://www.hfgproject.org/wp-content/uploads/2015/10/HFG-Mobile-Money-Compendium_October-2015.pdf (Accessed June 11, 2015).

[8] Mugisha D. Evolution of the payments industry in Uganda Pricewater Coopers (PwC) Uganda. (2022). Available online at: www.pwc.com/structure/

[9] WHO. WHO Guideline on Health Policy and System Support to Optimize Community Health Worker Programmes. Geneva: World Health Organization (2018). ISBN 978-92-4-155036-9. 30431747

[10] Yehualashet YG Wadda A Agblewonu KB Zhema T Ibrahim A Corr A 2016 World health organization’s innovative direct disbursement mechanism for payment of grassroots immunization personnel and operations in Nigeria: 2004–2015. J Infect Dis. (2016) 213:S108–15. 10.1093/infdis/jiv485 26908746 PMC4818546

[11] Zheng C Musominali S Chaw GF Paccione G. A performance-based incentives system for village health workers in Kisoro, Uganda. Ann Glob Health. (2019) 85(1):46, 1–9. 10.5334/aogh.2400 30924618 PMC6634603

[12] Rohatgi S Galdava E MBale A. The Role of Digital Financial Services in Accelerating USAID’s Health Goals. (2018). Available online at: https://www.usaid.gov/sites/default/files/documents/15396/DFS_Accelerating_USAID_HealthGoals.pdf (Accessed June 9, 2023).

[13] Sharp C Shuayb M Judkins M Hetherington M. Using Appreciative Inquiry in Educational Research: Possibilities and Limitations. Report. United Kingdom: National Foundation for Educational Research (2009). Available online at: https://www.nfer.ac.uk/media/1570/aen01.pdf

[14] Mangone E Riley P Datari K. Digital Financial Services for Health: A Global Evidence Review. (2021). Available online at: https://pdf.usaid.gov/pdf_docs/PA00XDJ7.pdf (Accessed June 9, 2023).

[15] O’Brien N Li E Chaibva C Bravo RG Kovacevic L Ayisi-Boateng NK Use of digital health technologies in primary health care (PHC) in the Sub Saharan Africa Region: a SWOT analysis (Preprint). (2022). Available online at: https://www.researchgate.net/publication/366828039 (Accessed December 15, 2022).

[16] Gronbach L. Social Cash Transfer Payment Systems in Sub-Saharan Africa: Centre for Social Science Research Working Paper 452. Cape Town: University of Cape Town (2020). Available online at: http://cssr.uct.ac.za/pub/wp/452

[17] Hemily D Castañeda CL Pattnaik A. A Framework for Purchasing in Community Health Worker Programs by “Last Mile Health Think Well”. (2021).

[18] Gentilini U Almenfi M Lyengar HT Okamura Y Downes JA Dale P Social Protection and Jobs Responses to COVID-19: A Real-Time Review of Country Measures. (2021).

[19] Wemos and Achest. Health workforce financing in Uganda: Country Report: Wemos Health Unlimited and African Center for Health and Social Transformation (Achest). (2019).

[20] Huang F Blaschke S Lucas H. Beyond pilotitis: taking digital health interventions to the national level in China and Uganda. Global Health. (2017) 13:49. 10.1186/s12992-017-0275-z 28756767 PMC5535287

[21] Egami H Matsumoto T. Mobile money use and healthcare utilization: evidence from rural Uganda. Sustainability. (2020) 12:3741. 10.3390/su12093741

[22] Cometto G Ford N Pfaffman-Zambruni Elie A Akl Uta Lehmann Barbara McPake Health policy and system support to optimise community health worker programmes: an abridged WHO guideline. Lancet Glob Health. (2018) 6:4001–2. 10.1016/S2214-109X(18)30482-0 30430994

[23] Ekirapa E Ashaba M Katamba P. Digital payment to community health workers in Uganda: lessons learnt from non-governmental organizations. (2022). Available online at: https://dhpir.mak.ac.ug/ (Accessed March 21, 2023).

[24] Muhangi K Mullengi B Babirye JK. Regulating Payment Systems in Uganda by KTA Advocates. (2022). Available online at: www.KTAAdvocate.com

[25] Ministère de la Santé Publique Bénin. Politique Nationale de la Santé Communautaire. Benin: Government of Benin (2015).

[26] Gouvernement de la République du Bénin. Réforme des pensions de retraite retraite: le paiement électronique devenu une réalité. (2020). Available online at: https://www.gouv.bj/actualite/923/reforme-pensions-retraite-paiement-electronique-devenu-realite/ (Accessed May 6, 2020).

[27] Secrétariat Général du Gouvernement de la République du Bénin. Adoption de la politique nationale de santé communautaire 2020-2024. Available online at: https://sgg.gouv.bj/cm/2020-05-06/download (Accessed May 6, 2020).

[28] Kallon I. La situation des agents de santé communautaires en Afrique: une étude présentant les documents disponibles sur les initiatives en matière de soins de santé primaires. (2020).

[29] AFI (Alliance for Financial Inclusion). Uganda’s journey to inclusive finance through digital financial services. (2019).

[30] Ormel H Kok M Kane S Ahmed R Chikaphupha K Rashid SF Salaried and voluntary community health workers: exploring how incentives and expectation gaps influence motivation. Hum Resour Health. (2019) 17(1):236–42. 10.1186/s12960-019-0387-z PMC6642499 31324192

[31] Bossé S Mercier S. Vitalizing the organization with the appreciative enquiry approach. Nutr Sci. (2019) 16(2):9.

[32] Holt DT Armenakis AA Feild HS Harris SG. Readiness for organizational change: the systematic development of a scale. J Appl Behav Sci. (2007) 43(2):232–55. 10.1177/0021886306295295

[33] Shea CM Jacobs SR Esserman DA Bruce K Weiner BJ. Organizational readiness for implementing change: a psychometric assessment of a new measure. (2014) 3:80–5. 10.1186/1748-5908-9-7 PMC3904699 24410955

[34] Weiner BJ. A theory of organizational readiness for change. Implement Sci. (2009) 4:67. 10.1186/1748-5908-4-67 19840381 PMC2770024

[35] Braun V Clarke V. Using thematic analysis in psychology. Qual Res Psychol. (2006) 3(2):77–101. 10.1191/1478088706qp063oa

[36] Sessions D Gatt L Gatabaki S. Digitizing Payments for USAID Beneficiaries in Uganda Pilot Report by Vital Wave. (2014).

[37] Soutter L Ferguson K Neubert M. Digital payments: impact factors and mass adoption in Sub-Saharan Africa. Technol Innov Manag Rev. (2019) 9(7):13–20.

[38] Hamani A Hussein Jama I Roland MAY Wanjeri L Oppon-Kusi AA Karimi D Mobile money and the importance of timely, complete payments to frontline health campaign workers in the fight to eradicate polio: pilot experience from a world health organization digital payment platform in Africa. BMC Health Serv Res. (2023) 23(1):16. 10.1186/s12913-022-08990-4 36611190 PMC9824973

